# Cerebellar GABA Levels and Cognitive Interference in Parkinson’s disease and Healthy Comparators

**DOI:** 10.3390/jpm11010016

**Published:** 2020-12-28

**Authors:** Federica Piras, Daniela Vecchio, Francesca Assogna, Clelia Pellicano, Valentina Ciullo, Nerisa Banaj, Richard A. E. Edden, Francesco E. Pontieri, Fabrizio Piras, Gianfranco Spalletta

**Affiliations:** 1 Neuropsychiatry Laboratory, Department of Clinical and Behavioral Neurology, IRCCS Santa Lucia Foundation, Via Ardeatina 306/354, 00179 Rome, Italy; federica.piras@hsantalucia.it (F.P.); d.vecchio@hsantalucia.it (D.V.); f.assogna@hsantalucia.it (F.A.); c.pellicano@hsantalucia.it (C.P.); v.ciullo@hsantalucia.it (V.C.); n.banaj@hsantalucia.it (N.B.); f.piras@hsantalucia.it (F.P.); 2 Department of Radiology, Kennedy Krieger Institute 707 North Broadway, Johns Hopkins University, Baltimore, MD 21205, USA; richardedden@gmail.com; 3 Department of Neuroscience, Mental Health and Sensory Organs (NESMOS), “Sant’Andrea” University Hospital, via di Grottarossa 1035-1037, 00189 Rome, Italy; fe.pontieri@gmail.com; 4 Menninger Department of Psychiatry and Behavioral Sciences, Baylor College of Medicine, 1977 Butler Blvd., Houston, TX 77030, USA

**Keywords:** Parkinson’s Disease, cognition, GABAergic signaling, cerebellum, MRS, response inhibition

## Abstract

The neuroanatomical and molecular substrates for cognitive impairment in Parkinson Disease (PD) are far from clear. Evidence suggests a non-dopaminergic basis, and a crucial role for cerebellum in cognitive control in PD. We investigated whether a PD cognitive marker (response inhibition) was differently controlled by g-amino butyric acid (GABA) and/or by glutamate-glutamine (Glx) levels in the cerebellum of idiopathic PD patients, and healthy comparators (HC). Magnetic resonance spectroscopy of GABA/Glx (MEGA-PRESS acquisition sequence) was performed at 3 Tesla, and response inhibition assessed by the Stroop Word-Color Test (SWCT) and the Wisconsin Card Sorting Test (WCST). Linear correlations between cerebellar GABA/Glx levels, SWCT time/error interference effects and WCST perseverative errors were performed to test differences between correlation coefficients in PD and HC. Results showed that higher levels of mean cerebellar GABA were associated to SWCT increased time and error interference effects in PD, and the contrary in HC. Such effect dissociated by hemisphere, while correlation coefficients differences were significant in both right and left cerebellum. We conclude that MRS measured levels of cerebellar GABA are related in PD patients with decreased efficiency in filtering task-irrelevant information. This is crucial for developing pharmacological treatments for PD to potentially preserve cognitive functioning.

## 1. Introduction

Converging evidence indicates that alterations in neurotransmitters beyond the dopamine system are present in Parkinson’s Disease (PD), and may disturb fronto-striatal related cognition [1]. Cognitive dysfunction is a well-known precocious non-motor manifestation of PD, and is indicative of risk of developing the disorder in subjects with predictive markers of the illness [2]. Particularly, loss of response inhibition, i.e., the ability to suppress a prepotent behavioral response, is a sensitive measure for diagnosis and progression of PD [3], linked to broader clinical deficits and predictive of later dementia. However, the neuroanatomical and molecular substrates for cognitive impairment in PD are far from clear. Rather than to frank neurodegeneration, cognitive dysfunctions in PD may also be attributable to dysregulation of non-dopaminergic neurotransmitter systems implicated in the disorder, the g-amino butyric acid (GABA) and glutamate (glutamate/glutamine complex: Glx) systems [1,4]. Concurrently, the role of the cerebellum in the pathophysiology of PD has been reconsidered [5], since it participates in compensatory mechanisms to delay symptom onset and to preserve optimal level of performance [6].

Based on the strong cerebellar-cortical interactions during information processing [7], we hypothesized that the cerebellum contributes to response inhibition performance. Therefore, we noninvasively probed, using magnetic resonance spectroscopy (MRS), the relationship between cerebellar GABA/Glx levels and response inhibition performance in a cohort of patients diagnosed with PD and in healthy comparators (HC). We predicted that cerebellar GABAergic signaling would be related in PD to response inhibition measures [1] and that changes in extracellular cerebellar GABA would explain variations in cognitive control efficiency.

## 2. Materials and Methods

### 2.1. Participants

In the present case-control correlational study, 25 consecutive subjects diagnosed with PD according to the UK Parkinson’s Disease Society Brain Bank diagnostic criteria [8] in the early stages (modified Hoehn and Yahr scale [9] ≤2) were initially selected for possible inclusion. All subjects were enrolled at the Movement Disorder Outpatient Services of our Institutions (IRCCS Santa Lucia Foundation; Department of Neuroscience, Mental Health and Sensory Organs, University “Sapienza”, Sant’Andrea Hospital) between January 2016 and January 2017. All patients were regularly followed-up in our outpatient clinics and recruited during scheduled visits. Clinical diagnosis of PD was confirmed along a follow-up period of 36-months from symptom onset.

Since there are no published data on the relationship between cerebellar GABA and Glx (Glutamate/Glutamine complex) levels and response inhibition abilities, either in PD or in HC, to determine a sufficient sample size for a two tailed z test on the difference between two independent correlations, a power analysis was conducted using an alpha of 0.05, a power of 0.80, and a very large effect size (Cohen’s *q* = 1) in order to detect only effects that would have clinical significance. Based on the aforementioned assumptions, the minimum number of necessary samples to meet the desired statistical constraints is 16 per group.

Dopamine replacement therapy dosages were calculated as daily levodopa equivalents. The following conversion table was applied: 100 mg levodopa = 1 mg pramipexole = 5 mg ropinirole = 5 mg rotigotine [10]. The levodopa equivalent dose of a drug is that which produces the same level of symptomatic control as 100 mg of immediate release L-dopa (taken with carbidopa) and expressed as the amount of levodopa that has a similar effect as the drug taken. The total daily levodopa equivalent dose (mg/day, see Table 1), obtained by adding together the levodopa equivalent dose for each antiparkinsonian drug, provides a summary of the total daily antiparkinsonian medication a patient is receiving. Out of the original group of patients confidently diagnosed with PD, 5 were excluded because were not able to complete the entire magnetic resonance exam, or because of the presence of artefacts or brain abnormalities (see exclusion criteria below). The remaining 20 patients included were age- and gender matched to 20 HC recruited through local advertisement in the same geographical area. HC and PD were screened for a current or lifetime history of DSM-5 mental and personality disorders using the SCID-5 -RV [11] and SCID-5-PD [12].

Inclusion criteria for all subjects were: (1) age between 18 and 65 years, (2) at least eight years of education, and (3) suitability for MRI scanning. Exclusion criteria were: (1) known or suspected history of alcoholism, drug dependence or abuse, other neurological disorders, (2) personality disorder, any present mental disorder (unipolar depressive and anxiety disorders of mild to moderate severity were suitable for recruitment) and past major mental disorders (however, a positive anamnesis for past unipolar mood and/or anxiety disorders of mild to moderate severity was considered acceptable for inclusion), according to DSM-5 criteria, (3) major medical illnesses, i.e., diabetes not stabilized, obstructive pulmonary disease or asthma, hematological/oncological disorders, B12 or folate deficiency as evidenced by blood concentrations below the lower normal limit, pernicious anemia, clinically significant and unstable active gastrointestinal, renal, hepatic, endocrine or cardiovascular system disease, newly treated hypothyroidism, (4) IQ below the normal range according to TIB (Test Intelligenza Breve, Italian analog of the National Adult Reading Test – NART) [13], (5) diagnosis of dementia according to the Movement Disorder Society clinical diagnostic criteria [14], (6) any potential brain abnormalities and vascular lesions as apparent on conventional T2- and FLAIR-scans; in particular, the presence, severity, and location of vascular lesions were rated according to the semi-automated method described elsewhere [15].

Sociodemographic characteristics, clinical features, and dopamine replacement therapy dosages for the PD group are summarized in Table 1.

### 2.2. Ethics Statement

The study was approved (by a written statement containing a waiver) and undertaken in accordance with the guidelines of the Santa Lucia Foundation Ethics Committee. All participants gave their written informed consent for research after they had received a complete explanation of the study procedures. Information about the potential publication of research results was included in the form, and a signed consent to the processing of personal data obtained from all participants.

### 2.3. Data Availability Statement

The batch-processing tool for the quantitative analysis of GABA-edited MR spectroscopy spectra used in this study is available for immediate download at https://github.com/richardedden/Gannet3.0/archive/master.zip. Due to a lack of consent of the participants, structural and chemical MRI data cannot be shared publicly, and can only be made available upon reasonable request if data privacy can be guaranteed according to the rules of the European General Data Protection Regulation (EU GDPR). The respective research group has to sign a data use agreement to follow these rules. This statement is in line with our institute’s policies and requirements by our funding bodies.

### 2.4. Neurological and Psychiatric Evaluation

Demographic and neurological features were collected at enrolment by a trained neurologist (CP or FEP) with expertise on parkinsonism. Disease stage was measured by the modified Hoehn and Yahr scale [9], and the severity of motor symptoms by the UPDRS-III scale [16]. Patients diagnosed with PD underwent a detailed neuropsychiatric evaluation. Apathy was diagnosed according to the adapted Marin’s criteria [17]. Severity of anxiety symptoms was quantified by the Hamilton Anxiety Rating Scale (HARS) (total score). Severity of depressive symptoms was investigated by the Beck Depression Inventory (BDI) (total score, psychic and somatic sub-scores). Apathy severity was quantified by means of the Apathy Scale (AS) (total score, motivation, interest, effort, indifference/lack of emotion). The Parkinson’s Psychosis Rating Scale (PPRS) (total, hallucinations, illusions, paranoid ideation, sleep disturbances, confusion, and sexual preoccupation sub-scores) was used to assess the severity of psychotic symptoms. Clinical interviews and mental disorder diagnoses were made by a senior psychiatrist (GS).

### 2.5. Cognitive Assessment

After having been screened for global cognitive impairment using the Mini-Mental State Examination (MMSE), all study subjects underwent the Mental Deterioration Battery (MDB) [18]. The latter was performed by two trained neuropsychologists (FeP and FaP) and administered to further exclude, by means of standardized cognitive testing, the presence of major neurocognitive disorder (i.e., scores lower than the tolerance level in at least two MBD tests [14]). Acceptable inter-rater reliability was defined as k > 0.80.

Details on methodology for neuropsychological and psychopathologic evaluations have been published elsewhere [19].

Two traditional set-shifting tests, i.e., the Modified Wisconsin Card Sorting Test short form (M-WCST-sf) [20], and the short version of the Stroop Word-Color Test (SWCT-sv) [21] were administered to explore response inhibition abilities. In these tasks subjects are required to attend to a particular property of a presented visual stimulus, and to select a feature-specific response. In the M-WCST-sf, participants are asked to sort 48 response cards to match either color (red, blue, yellow, or green), form (crosses, circles, triangles, or stars), or number (one, two, three, four) of four stimulus figures. They are expected to accurately sort every response card according to one of three possible sorting criteria, through the feedback (right or wrong) given by the examiner. During the task, the sorting rule changes discreetly from color to form to number of figures, without the participants being informed. Participants have to shift sets accordingly, and to detect the new valid rule by a trial and error procedure. Achieved categories (C), perseverative (P) and non-perseverative (NP) errors were calculated. Set shifting and response inhibition difficulties are indicated by perseverative errors; thus, higher scores represent worse performance. The SWCT-sv comprises three subtests: “word reading” (W), “color naming” (C) and “word-color interference” (I). In the latter, the different stimulus properties interfere with each other since written colored words serve as stimulus displays and participants are instructed to switch between the response rules “color naming” and “word reading.” The valid response rule is indicated by an explicit task cue. Response inhibition abilities were evaluated by computing a time interference effect (based on execution time) and an error interference effect (based on number of errors) [21]. Neuropsychological and neuropsychiatric scores are shown in Table 1. Neuropsychological data were collected within 2 days from MRI scanning.

### 2.6. MRS Acquisition and Processing

Magnetic resonance scanning was conducted on a Philips 3.0 T Achieva system with a 32 channel receiving only head coil (Philips Medical Systems, Best, The Netherlands). Head position was fixed with foam padding to minimize movements.

**Figure 1:**
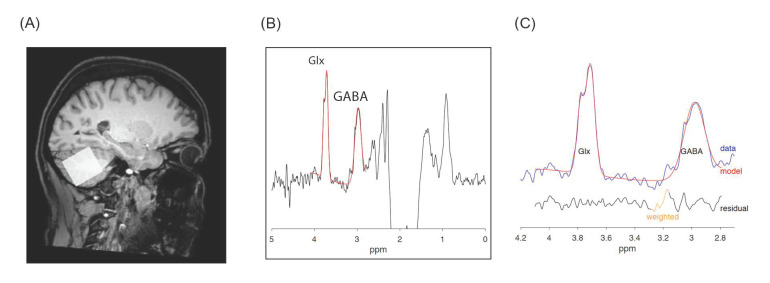
Cerebellar GABA and Glx spectra. Legend: (**A**) Voxel placement in the right hemisphere; (**B**) acquired Magnetic Resonance Spectroscopy (MRS) spectra with gamma-aminobutyric acid (GABA) and Glutamate/Glutamine complex (Glx) peaks in red, and (**C**) zoom on GABA and Glx peaks: acquired data are in blue, fit in red and residual in black.

T1-weighted structural magnetic resonance images were acquired for spectroscopic voxel placement (TR = 300–500 ms, TE = 5.3 msec, matrix = 256 × 228, FOV = 230 × 233, slice thickness = 0.9 mm, flip angle = 8°). T2 and FLAIR sequences were acquired to clinically screen for possible brain pathology. GABA and Glx measurements in the left and right cerebellar hemispheres were obtained using the MEGA-PRESS acquisition sequence (TR = 2.0 s; TE = 68ms; 14 ms editing pulse applied at 1.9 ppm and 7.5 ppm, 256 averages, voxel size 3 × 3 × 3 cm^3^), an efficient and reliable sequence for detecting brain level of endogenous GABA [22] and other brain metabolites. Voxel size was sufficient to include each cerebellar hemisphere; all voxels were positioned in the subjects’ native space to minimize the signal coming from cerebrovascular fluid (CSF) and skull. Figure 1 shows the location of voxel in the right cerebellum, a typical GABA, Glx MEGA-PRESS spectrum and the fitted GABA, Glx peaks.

Quantification was performed using the Gannet 3.0 toolkit (Baltimore, MD, USA), a Matlab-based quantitative batch analysis tool specifically developed for GABA MEGA-PRESS spectra [23]. Gannet contains five modules: GannetLoad, GannetFit, GannetCoRegister, GannetSegment and GannetQuantify. The GannetLoad module is used to parse certain variables from the data headers, apply a line broadening of 3 Hz, and frequency and phase correct the individual spectra using Spectral Registration. GannetFit uses a single-Gaussian model to fit the edited GABA and Glx signals and evaluates both metabolites relative to creatine (Cr). GannetCoRegister takes location and orientation information from the headers of MRI and image data, and generates a binary mask representing the voxel location in the matrix of the image. GannetSegment calls an SPM segmentation of the T1-weighted anatomical image and reports the tissue fractions of the voxel mask generated by GannetCoRegister. GannetQuantify combines modelled peak areas from GannetFit and voxel tissue fractions from GannetSegment with preset values for GABA and Glx and water relaxation and visibility, to deliver concentration values. In order to address differences in GABA and Glx levels of the different tissue compartments that make up the MRS voxel, metabolites concentration was quantified relative to the unsuppressed water signal, corrected for voxel tissue composition (voxel fractions of white, grey matter and cerebrospinal fluid, i.e., alpha-correction).

### 2.7. Statistical Analysis

PD and HC were first compared in terms of sociodemographic characteristics (age, education level and gender), and cerebellar GABA, Glx (total glutamate+glutamine, as a measure of excitatory function) levels, and Glx/GABA ratios (computed as left, right and mean (left+right/2) cerebellar Glx levels divided by GABA levels to assess the excitation/inhibition balance in the voxel) using chi-square and unpaired t-tests. Paired t-tests were used to compare cerebellar GABA, Glx levels and Glx/GABA ratios between hemispheres within diagnostic groups. In PD, linear correlations (significance was tested by Fisher’s r to z transformation) between left, right and mean (left+right/2) GABA, Glx levels and Glx/GABA ratios and dopamine replacement therapy dosages (expressed as daily levodopa equivalents) were computed to verify potential medication effects on GABA and Glx signals measured by MRS [4]. Equally, given the intertwined relationship between dopaminergic replacement therapy and psychiatric symptoms phenomenology in PD, and considering psychosis as a possible medication side effect [24], the correlations between daily levodopa equivalents and AS, HARS, BDI, and PPRS total scores were evaluated. The same psychiatric measures were correlated to M-WCST-sf (perseverative errors, M-WCST-sf P) and SWC-sv (interference effect-time, SWC-sv IE-T, interference effect-errors, SWC-sv IE-E) performance in order to explore the pattern of potential interactions between psychopathology and response inhibition abilities. Whenever significant, correlating factors were used as covariates in partial correlation analyses in order to confirm the strength and direction of the linear relationship between the two random variables, with the effect of a controlling random variable removed.

Linear correlations between cerebellar GABA, Glx levels, Glx/GABA ratios and response inhibition performance, separately for PD and HC, were performed for the M-WCST-sf P, the SWC-sv IE-T and the SWC-sv IE-E. To provide a direct test of a model assuming a different relationship in PD and HC between cerebellar GABA, Glx levels, Glx/GABA ratios and response inhibition performance, the significance of a potential difference between correlation coefficients in the two groups was tested. This was calculated as follows:

$$Z\;\mathrm{observed}=(z_{1}\;-z_{2})÷(\sqrt{\left[\left(1÷N_{1}-3\right)+\left(1÷N_{2}-3\right)\right]}$$

where z_1_ and z_2_ are the Fisher’s transformed values of the two correlations and N_1_, N_2_ the respective sample size. Significance of the z test was set at *p* < 0.01 (two tailed) after correction for multiple comparisons (0.05/3 response inhibition scores).

## 3. Results

### 3.1. Sociodemographic, Neuropsychiatric and Cognitive Features

The two diagnostic groups did not differ for age, gender, and educational attainment. Within the PD patients’ cohort, one patient (5%) met diagnostic criteria for apathy [17]. As for mood disorders, two patients (10%) met the DSM 5 criteria for major depressive disorder, while 30% (*n* = 6) was diagnosed with a depressive disorder not otherwise specified. Severity of anxiety and depressive symptoms is reported in Table 1. No PD patient met DSM 5 criteria for psychosis (see Table 1 for PPRS total and sub-scores). Although PD patients significantly differed from HC respect to MMSE total score, no study subject met a formal diagnosis of major neurocognitive disorder [14]. The two groups significantly differed respect to number of perseverative errors in the M-WCST-sf, with a borderline significant trend for the SWC-sv error interference effect (see Table 1).

A significant negative correlation in the patients’ group was observed between HARS total score and daily levodopa equivalents (see Table 2). No other significant correlation was observable in PD patients between any neuropsychiatric tests scores and response inhibition abilities as indexed by the M-WCST-sf P, the SWC-sv IE-T and the SWC-sv IE-E scores.

### 3.2. Cerebellar GABA, Glx Levels and Excitation/Inhibition Balance

The cerebellar metabolite levels and their excitation/inhibition balance did not differ between PD and HC (sees Table 1). Concentrations of GABA, Glx and excitation/inhibition balance did not differ between right and left cerebellar hemispheres in either diagnostic group.

In PD, mean cerebellar Glx concentration was correlated with daily levodopa dosages, with a tendency toward significance for the correlation between the latter and Glx level in the right cerebellar hemisphere (see Table 3). No other significant correlation between daily levodopa dosages and cerebellar metabolite concentrations was observed.

**Figure 2:**
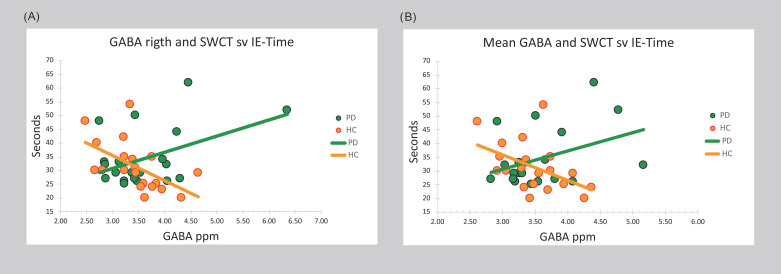
Bivariate scattergrams of the differential relationship in experimental groups between cerebellar gamma-aminobutyric acid (GABA) and cognitive interference. Legend: (**A**) right and (**B**) left and right mean values of GABA cerebellar concentration and response inhibition performance in patients diagnosed with Parkinson Disease (PD) and Healthy Comparators (HC), as measured through the time (in seconds) Interference Effect of the Stroop Word-Color Test short version (SWCT sv IE-Time). ppm: parts per million.

In HC, mean cerebellar GABA level positively correlated with the SWCT-sv IE-T score (see Table 3 and Figure 2). In PD, a positive correlation was observed between mean GABA level and the SWCT-sv IE-E score. Focusing on hemispheres, a positive significant correlation was detected between left cerebellar GABA level and the SWCT-sv IE-E score in the PD cohort, while right cerebellar GABA level negatively correlated with the SWCT-sv IE-T score in HC, and positively in PD (see Table 3 and Figure 2).

No significant correlations were observed between mean, left and right Glx levels and neuropsychological measures indexing response inhibition abilities. However, given the significant correlation between daily levodopa dosage and mean cerebellar Glx level, and in an attempt to correct for this effect, partial correlations were calculated between the latter and WSCT-sf P, SWCT-sv IE-T and SWCT-sv IE-E scores in PD. Again, no significant correlation emerged when the effect of dopamine replacement treatment was removed, apart from a tendency toward significance for the relationship between the WSCT-sf P score and mean cerebellar Glx levels (r = 0.40; *p* = 0.08). A positive significant correlation was observed in HC between the SWCT-sv IE-T score and the excitation/inhibition balance in the right cerebellar hemisphere. No other significant correlation was present in both groups between the measured cerebellar metabolites, the excitation/inhibition balance within the cerebellar voxel and scores indexing response inhibition abilities.

### 3.3. Comparisons between PD and HC Correlation Coefficients

After FDR correction for multiple comparisons, the difference between HC and PD correlation coefficients was significant for the interdependence between mean cerebellar GABA level and the SWCT-sv IE-T and IE-E scores (negative in HC and positive in PD) and between the SWCT-sv IE-E score and left cerebellar GABA levels (negative in HC and positive in PD). A significant difference in correlation coefficients was also observed for the relationship between right GABA levels and the SWCT-sv IE-T score (negative in HC and positive in PD) (see Table 3).

## 4. Discussion

Here the role of cerebellar tonic inhibition in cognitive functioning was investigated under a pathological clinical condition and compared to what observed under normal physiological condition. Specifically, the correlation between GABA and Glx cerebellar levels (as measured by MRS) [25] and proper response inhibition (as typically assessed through time and error interference effects in the Stroop test) [20] was tested in a cohort of non-demented patients with PD and in HC. We found that while in PD patients increased GABAergic tonic inhibition in the cerebellum was associated with decreased efficiency in filtering task-irrelevant information, the reverse correlation was observable in HC.

Such results crucially demonstrate, in the first place, that the GABAergic neurochemical profile in the cerebellum is linked to response inhibition in both HC and patients diagnosed with PD. Since response inhibition is one of the most sensitive measures for characterizing the cognitive phenotype in PD [3], our results critically confirm the non-dopaminergic basis of this key cognitive deficit [26]. This extends to PD patients previous findings demonstrating that aberrant GABAergic inhibitory regulation of prefronto-cerebellar circuits underlies impairments in executive control [27].

Our results also substantiate that the cerebellum is a critical node in the distributed neural circuits subserving cognition [7] in PD also [28], and that this region should be increasingly recognized as being involved in the pathophysiology of the disorder [5]. They finally suggest that MR spectroscopic assessment of cerebellar GABA in PD may be a potential biomarker [29] in those patients showing changed performance in executive functioning tests [30].

Although the role of persistent cerebellar GABAergic inhibition in shaping brain function has been intensively studied, evidence is limited to animal studies and the motor and learning domains. Mediated through an activation of extrasynaptic GABA_A_ receptors by the tonically released GABA, tonic inhibition exerts a powerful action in cognitive functions by controlling neuronal excitability. Since it enforces a dynamic control of motor coordination [31] regulating the rate, rhythm and accuracy of movements, so it may also regulate the speed, capacity and appropriateness of mental and cognitive processes.

Indeed, the cerebellum is thought to mediate cortical information processing via closed cortico-cerebellar loops [32]. The unique connections to different areas of the cortex suggest that it may be involved in the executive control processes performed by the lateral prefrontal cortex (PFC) [33]. While the PFC sends signals to posterior portions of the brain to bias relevant over irrelevant information, cerebellar GABA-dependent tonic inhibition regulates sensory information transmission across the cerebellar cortex [34]. Thus, this mechanism may participate to the PFC-based enhancement of task-relevant information processing [35]. The here reported correlation between cerebellar GABA levels and response inhibition both in PD patients and HC, strongly supports this hypothesis suggesting that a balance of neurotransmitter activity in the cerebellum [34] regulates the gating of sensory information in the PFC. The observed hemispheric dissociation further supports this assumption. Indeed, GABA-dependent inhibition in the left cerebellar hemisphere correlated with the error interference effect in PD. Concurrently, tonic inhibition in the right cerebellar hemisphere was related to the time interference effect in both PD patients and HC. Contralateral cerebellar-cerebral connections with the PFC possibly exploited the reported correlations, as the left PFC is responsible for resolving semantic conflict, and the right PFC for response conflict [36].

However, we found that the relationship between cerebellar GABA-dependent tonic inhibition and response inhibition was reversed in PD patients. Actually, potential changes in cerebellar output in PD are still largely unknown. Indirect evidence suggests some functional changes in the cerebellar-cerebral circuitry, which may support compensatory mechanisms to the basal ganglia dysfunction [5,37]. Indeed, the here observed reversed association between cerebellar GABA-dependent tonic inhibition and preserved response inhibition in PD patients suggests the potential enactment of some kind of compensation to support optimal levels of performance [6]. For example, executive dysfunction in PD revolves around prefrontal dopamine systems [38]. Response inhibition in particular, evokes dopamine release in the PFC of HC [39], and dopamine pharmacological manipulation improves response inhibitory behavior [40] in PD patients also [41]. Animal studies demonstrate that cerebellar Purkinje cells output can modulate dopamine efflux in the PFC [42]. Thus, changes in cerebellar GABAergic transmission may be compensatory mechanisms for counteracting cognitive impairment associated with prefrontal dopaminergic dysfunction in PD. Alternatively, variations in cerebellar GABA-dependent tonic inhibition may compensate for the down-regulation of inhibitory neurotransmission in the frontal cortex observed in PD, as also suggested by molecular studies [43,44]. Since the level of inhibition is critically important for creating the attentional set that facilitates the selection of task-relevant representations in the Stroop task, it is clear that efficient inhibitory neurotransmission in the PFC is crucial for optimal performance [45]. Additionally, although the cerebellum receives mainly noradrenergic and serotonergic projections, there is also evidence for dopamine. The cerebellar cortex contains a high density of dopamine receptors, thus implying that cerebellar output may be affected by dopamine depletion in PD, but also by dopamine replacement therapy. Indeed, levodopa increases cerebellar activity restoring it to that observed in healthy controls in PD patients on medication after overnight withdrawal of dopaminergic replacement therapy [46]. Therefore, the here observed correlation between cerebellar GABA levels and normal response inhibition in PD might be indirectly related to medication status, and to the levodopa-related boost in activity in the basal ganglia (and the cerebellum) owing to the direct connections and enhanced connectivity after medication [47], between these structures. However, although preliminary given the case-control cross-sectional nature of the present finding, the observation that cerebellar metabolite levels and their excitation/inhibition balance were not correlated to dopamine replacement therapy dosages would suggest that both a potential dopamine depletion effect on cerebellar output and a possible indirect effect of medication status on cerebellar metabolite levels are unlikely.

Nevertheless, the reported inverted association (respect to what observed in HC) in PD patients may also be a disease-related change. It is, indeed, possible that GABA concentrations in the cerebellum are increased in the early stages of the disorder. This change would be a consequence of dopamine depletion in the basal ganglia, and related to the executive dysfunctions observed in early PD [1]. The evidence of cerebellar microstructural changes and GABA-related neuronal dysfunctions in the tremor-dominant subtype of PD [48,49,50] further suggests that such abnormalities may lead to pathologic activity along the cerebellar-cortical pathways. Yet, in our sample of medicated patients, both cerebellar GABA levels and response inhibition performance were not different from that of HC. Although dopamine replacement medication may have “normalized” cerebellar tonic inhibition and cognitive performance [41] in PD patients, no relationship was observed between daily levodopa-equivalent dosages and cerebellar GABA levels. Moreover, although within a very limited sub-sample (only four patients), cerebellar GABA levels in unmedicated PD subjects were always within the range (+/- 2 standard deviations from mean) of those measured in medicated PD patients. In contrast, a significant negative association was found in PD patients between mean cerebellar Glx levels and levodopa-equivalent dosages suggesting that glutamate levels were modulated by dopamine replacement therapy. However, Glx levels in the small sub-sample of unmedicated PD patients were comparable to Glx concentrations in the medicated sample. Future studies comparing groups of medicated and unmedicated patients or with a longitudinal approach, either within the same patients, or with patients at different levels of disease duration/progression will further clarify this issue. 

Actually, a first limitation of the present study is that the investigation of the relationship between GABA/Glx levels in the cerebellum and cognition in a sample of medicated PD patients may be influenced by the treatment itself. However, while dopamine replacement therapy was negatively correlated to mean cerebellar Glx levels, Glx did not affect response inhibition performance. Thus, the intervening effect of treatment on our main result is unlikely, also considering the null difference in cerebellar metabolite levels and their excitation/inhibition balance between medicated patients and the small sub-sample of unmedicated PD patients. Second, it might be argued that MRS cannot distinguish between synaptic and intracellular stores of GABA, thus impeding a detailed and definitive interpretation of the neurophysiological significance of our findings. Nevertheless, according to recent consensus, MRS is most sensitive to extracellular unbound GABA, which is involved in tonic inhibition [25]. Since such local tonic inhibition was related to cognitive efficiency in counteracting interference in both samples, we assumed that extracellular cerebellar GABA, which MRS can measure, participated to response inhibition. We therefore interpreted our findings at the macro-circuit system level postulating that a change in GABAergic neurotransmission within cerebellar-cerebral networks, served to maintain an optimal level of performance in PD patients [5]. Future studies measuring extracellular GABA in more than one cortical region within the executive control network (cerebellum included) will contribute to clarify the dynamic of cerebellar-cerebral networks and their relation with cognition. Additionally, the potentiality for MR spectroscopic assessment of cerebellar GABA in PD as a diagnostic biomarker should be further investigated [29]. Given the putative role of the cerebellum in the pathophysiology of the disorder, and the here reported relationship between cerebellar GABAergic signaling and measures for diagnosis and progression of PD [3], the neurochemical profile in the cerebellum might constitute an additional diagnostic marker [29] of the disorder.

A further potential limitation of the present study is the relatively small sample size, which might have increased the risk for type I error. However, a very large effect (Cohen’s q > 0.90) was observed for the difference in HC and PD correlation coefficients between mean cerebellar GABA levels and response inhibition performance, thus suggesting that the analyses were not underpowered (post-hoc computed beta = 0.80). Nevertheless, a further study including a larger sample is warranted also considering the significant (although not surviving to multiple comparisons) test of difference between HC and PD correlation coefficients between the excitation/inhibition balance in the cerebellum and measures of response inhibition.

## 5. Conclusions

Here we demonstrate for the first time, that cognitive efficiency in counteracting interference is related to GABA-dependent tonic inhibition in the cerebellum both under physiological and pathological conditions. We also underline that increased tonic inhibition in the cerebellum, relative to response inhibition, is associated to normal levels of performance in PD patients. Given the cross-sectional nature of the present study we could not establish the potential longitudinal “cost” of the observed change in the relationship between GABAergic neurotransmission and cognition in PD. However, our finding provides strong evidence that the cerebellum should be considered as a primary site for systems-level compensation in the disorder [5]. Considering the neuroprotective role of GABAergic inhibition [51], future intervention studies are necessary to test how the modulation of GABAergic mechanisms changes PD cognitive symptoms, and to establish whether GABA plays a compensatory or pathophysiological role in Parkinson’s disease. This is of clinical relevance since pharmacologically boosting GABAergic neurotransmission in PD patients modulates aberrant neuronal network oscillations at beta frequency [52], which seems to restore cognitive functions, at least in stroke patients.

## Figures and Tables

**Table 1:** Sociodemographic, clinical, psychopathological-cognitive characteristics, cerebellar GABA, Glx levels and excitation/inhibition balance in the studied samples.

Characteristics (Standard Deviation)	HC (*n* = 20)	PD (*n* = 20)	t or χ^2^	d.f.	*p*
Age (years/sd)	54.25 (16.62)	58.55 (9.6)	1.09	38	0.27
Males *n* (%)	12 (60)	10 (50)	0.4	1	0.52
Educational level (years/sd)	14.00 (3.21)	12.65 (3.97)	−1.18	38	0.24
Age at onset (years/sd)	-	55.4 (9.59)	-	-	-
Duration of illness (years/sd)	-	3.51 (1.78)	-	-	-
H&Y score	-	1.47 (0.47)	-	-	-
UPDRS-III score (sd)	-	12.30 (6.01)	-	-	-
Levodopa equivalents (mg/day-sd)	-	335.0 (260.99)	-	-	-
Combined dopamine agonists/levodopa treatment *n* (%)	-	7 (35)	-	-	-
Dopamine agonists monotherapy *n* (%)	-	6 (30)	-	-	-
Levodopa monotherapy *n* (%)	-	3 (15)	-	-	-
non medicated	-	4 (20)	-	-	-
Apathy diagnosis *n* (%)	-	1 (5)	-	-	-
AS tot. (score/sd)	-	8.75 (5.38)	-	-	-
AS motivation	-	0.60 (0.68)	-	-	-
AS interest	-	1.65 (1.66)	-	-	-
AS effort	-	0.45 (0.75)	-	-	-
AS indifference	-	0.65 (1.22)	-	-	-
HARS tot. (score/sd)	-	7.25 (4.54)	-	-	-
BDI tot. (score/sd)	-	9.10 (6.62)	-	-	-
BDI psychic	-	5.45 (4.51)	-	-	-
BDI somatic	-	3.65 (2.34)	-	-	-
PPRS tot. (score/sd)	-	6.55 (0.82)	-	-	-
PPRS hallucinations	-	1.10 (0.30)	-	-	-
PPRS illusions	-	1.05 (0.22)	-	-	-
PPRS paranoid ideation	-	1.0 (0.0)	-	-	-
PPRS sleep disturbance	-	1.30 (0.57)	-	-	-
PPRS confusion	-	1.0 (0.0)	-	-	-
PPRS sexual preoccupation	-	1.10 (0.45)	-	-	-
MMSE (raw score/sd)	29.50 (1.0)	28.80 (1.05)	2.15	38	0.04
M-WCST-sf C	5.95 (0.22)	5.95 (0.22)	0.0	38	1.0
M-WCST-sf P	0.15 (0.36)	1.0 (1.77)	-2.09	38	0.04
M-WCST-sf NP	0.60 (0.82)	1.0 (0.97)	−1.40	38	0.38
SWCT-sv IE-T (sec/sd)	31.40 (9.01)	34.55 (10.66)	−1.0	38	0.43
SWCT-sv IE-E	0.20 (0.69)	0.45 (1.05)	−0.88	38	0.09
Mean cerebellar GABA (ppm/sd)	3.48 (0.46)	3.59 (0.61)	−0.60	38	0.55
Cerebellar GABA left	3.53 (0.56)	3.55 (0.76)	−0.11	38	0.91
Cerebellar GABA right	3.44 (0.54)	3.62 (0.82)	−0.82	38	0.41
Mean cerebellar Glx	10.17 (0.99)	10.21 (1.0)	−0.12	38	0.90
Cerebellar Glx left	10.29 (1.31)	10.42 (1.16)	−0.35	38	0.73
Cerebellar Glx right	10.05 (1.29)	9.99 (1.34)	0.14	38	0.89
Mean cerebellar E/I balance (Glx/GABA)	2.97 (0.48)	2.91 (0.56)	0.31	38	0.75
Cerebellar E/I balance (Glx/GABA) left	2.99 (0.60)	3.04 (0.63)	−0.26	38	0.80
Cerebellar E/I balance (Glx/GABA) right	2.99 (0.60)	2.87 (0.70)	0.56	38	0.58

Legend: AS, Apathy Scale; BDI, Beck Depression Inventory; d.f., degree of freedom; E/I excitation/inhibition; GABA, gamma-aminobutyric acid; Glx, Glutamate/Glutamine complex; HC, healthy controls; H&Y, Hoehn and Yahr scale; HARS, Hamilton Anxiety Rating Scale; HC, healthy controls; M-WCST-sf, Modified Wisconsin Card Sorting Test short form; M-WCST-sf C achieved categories; M-WCST-sf NP non-perseverative errors; M-WCST-sf P perseverative errors; MMSE, Minimental State Examination; PD, Parkinson Disease patients; ppm, parts per million; PPRS, Parkinson’s Psychosis Rating Scale; SWCT-sv IE-E, Stroop Word-Color Test short form error interference effect; SWCT-sv IE-T, Stroop Word-Color Test short form time interference effect; UPDRS-III scale, Unified Parkinson’s Disease Rating Scale Part III motor function. Bold values indicate statistically significant differences.

**Table 2:** Correlations in Parkinson’s Disease patients, between neuropsychiatric tests scores, dopamine replacement therapy and response inhibition tests scores.

	Levodopa eq. r to z (*p level*)	M-WCST-sf P r to z (*p* level)	SWCT-sv IE-T r to z (*p* level)	SWCT-sv IE-E r to z (*p level*)
AS tot	−0.06 (0.81)	0.14 (0.55)	0.10 (0.68)	0.16 (0.50)
HARS tot	**−0.52 (0.02)**	−0.11 (0.64)	−0.16 (0.49)	0.06 (0.79)
BDI tot	−0.13 (0.58)	0.03 (0.90)	−0.04 (0.85)	−0.11(0.64)
PPRS tot	0.37 (0.10)	0.04 (0.88)	−0.06 (0.80)	0.25 (0.30)

Legend: AS, Apathy Scale; BDI, Beck Depression Inventory; HARS, Hamilton Anxiety Rating Scale; M-WCST-sf, P Modified Wisconsin Card Sorting Test short form perseverative errors; PPRS, Parkinson’s Psychosis Rating Scale; r to z, Fisher’s r to z transformation; SWCT-sv IE-E, Stroop Word-Color Test short form, error interference effect; SWCT-sv IE-T, Stroop Word-Color Test short form, time interference effect. Daily levodopa equivalent doses are expressed as mg/day. Bold values indicate statistically significant differences.

**Table 3:** Correlations between cerebellar GABA, Glx levels, excitation/inhibition balance and dopamine replacement therapy (daily levodopa equivalents), response inhibition measures in the studied samples and results from the test of difference between correlation.

	**Mean GABA** **r to z (*p* level)**			**GABA left** **r to z (*p* level)**			**GABA right** **r to z (*p* level)**		
	HC = 20	PD = 20	Z-test z (*p* level)	HC = 20	PD = 20	Z-test z (*p* level)	HC=20	PD = 20	Z-test z (*p* level)
levodopa eq. (mg/day)	-	0.007 (.98)	-	-	0.06 (0.80)	-	-	−0.05 (0.85)	-
M-WCST-sf P	0.05 (0.82)	0.31 (0.19)	−0.79(ns)	0.11 (0.64)	0.11 (0.63)	0 (ns)	−0.03 (0.91)	0.35 (0.12)	−1.15(ns)
SWCT-sv IE-T	**−0.47 (0.03)**	0.39 (0.09)	−2.69 (0.007) *	−0.25 (0.29)	0.13 (0.59)	−1.13 (ns)	**−0.55 (0.01)**	**0.45 (0.04)**	−3.22 (0.001) *
SWCT-sv IE-E	−0.42(0.06)	**0.48 (0.03)**	−2.83 (0.005) *	−0.30 (0.20)	**0.53 (0.01)**	−2.62 (0.009) *	−0.40 (0.08)	0.21 (0.36)	−1.86 (ns)
	**Mean Glx** **r to z (*p* level)**			**Glx left** **r to z (*p* level))**			**Glx right** **r to z (*p* level))**		
	HC = 20	PD = 20	Z-test z (*p* level)	HC = 20	PD = 20	Z-test z (*p* level)	HC = 20	PD = 20	Z-test z (*p* level)
levodopa eq. (mg/day)	-	**−0.50 (0.02)**	-	-	−0.36 (0.11)	-	-	−0.43 (0.06)	-
M-WCST-sf P	0.06 (0.80)	0.31 (0.18)	−0.76 (ns)	−0.28 (0.24)	0.21 (0.36)	−1.46 (ns)	0.37 (0.10)	0.28 (0.24)	0.29 (ns)
SWCT-sv IE-T	0.07 (0.78)	0.09 (0.71)	−0.06 (ns)	−0.002 (0.1)	−0.01 (0.96)	0.02 (ns)	0.10 (0.67)	0.14 (0.54)	−0.12 (ns)
SWCT-sv IE-E	−0.18 (0.44)	0.30 (0.19)	−1.43 (ns)	−0.25 (0.28)	0.06 (0.79)	−0.92 (ns)	−0.02 (0.94)	0.40 (0.08)	−1.29 (ns)
	**Mean E/I (Glx/GABA)** **r to z (*p* level))**			**E/I (Glx/GABA) left** **r to z (*p* level))**			**E/I (Glx/GABA) right** **r to z (*p* level))**		
	HC = 20	PD = 20	Z-test z (*p* level)	HC = 20	PD = 20	Z-test z (*p* level)	HC = 20	PD = 20	Z-test z (*p* level)
levodopa eq. (mg/day)	-	−0.29 (0.21)	-	-	−0.32 (0.17)	-	-	−0.21 (0.37)	-
M-WCST-sf P	−0.03 (0.89)	−0.12 (0.61)	0.26 (ns)	−0.28 (0.23)	−0.06 (0.79)	−0.66 (ns)	0.25 (0.29)	−0.13 (0.58)	1.13 (ns)
SWCT-sv IE-T	0.40 (0.07)	−0.28 (0.23)	**2.07 (0.03)**	0.13 (0.59)	−0.19 (0.42)	0.94 (ns)	**0.51 (0.02)**	−0.22 (0.35)	**2.29 (0.02)**
SWCT-sv IE-E	0.27 (0.25)	−0.22 (.36)	1.46 (ns)	0.06 (0.81)	−0.35 (0.13)	1.24 (ns)	0.37 (0.10)	−0.03 (0.90)	1.22 (ns)

Legend: HC, healthy controls; E/I excitation/inhibition; GABA, gamma-aminobutyric acid; Glx, Glutamate/Glutamine complex; M-WCST-sf P, Modified Wisconsin Card Sorting Test short form perseverative errors; ns, non-significant; PD, Parkinson disease patients; r to z, Fisher’s r to z transformation; SWCT-sv IE-E, Stroop Word-Color Test short form, error interference effect; SWCT-sv IE-T, Stroop Word-Color Test short form, time interference effect. *Significant after correction for multiple comparisons (i.e., *p* < 0.01 see text for reference). Bold values indicate statistical significance.
